# Risk factors and implications of progressive coronary dilatation in children with Kawasaki disease

**DOI:** 10.1186/s12887-017-0895-8

**Published:** 2017-06-06

**Authors:** Ming-Yu Liu, Hsin-Min Liu, Chia-Hui Wu, Chin-Hao Chang, Guan-Jr Huang, Chun-An Chen, Shuenn-Nan Chiu, Chun-Wei Lu, Ming-Tai Lin, Luan-Yin Chang, Jou-Kou Wang, Mei-Hwan Wu

**Affiliations:** 1 Department of Pediatrics, National Taiwan University Hospital and Medical College, National Taiwan University, No. 7, Chung-Shan South Road, Taipei, 100 Taiwan; 2 Department of Medical Research, National Taiwan University Hospital, Taipei, Taiwan; 3 Medical Information Management Office, National Taiwan University Hospital, Taipei, Taiwan

**Keywords:** Kawasaki disease, Risk factors, Progressive coronary dilatation, Hypoalbuminemia

## Abstract

**Background:**

Kawasaki disease (KD) is an acute systemic vasculitis that occurs in children and may lead to cardiovascular morbidity and mortality. Progressive coronary dilatation for at least 2 months is associated with worse late coronary outcomes in patients with KD having medium or giant aneurysms. However, the risk factors and occurrence of progressive coronary dilatation in patients with KD but without medium or giant aneurysms have been insufficiently explored.

**Methods:**

We retrospectively enrolled 169 patients with KD from a tertiary medical center in Taiwan during 2009–2013. Medical records of all patients were reviewed. Echocardiography was performed during the acute KD phase and at 3–4 weeks, 6–8 weeks, 6 months, and 12 months after KD onset. Progressive coronary dilatation was defined as the progressive enlargement of coronary arteries on three consecutive echocardiograms. Logistic regression analysis was conducted to evaluate the potential risk factors for coronary aneurysms and progressive coronary dilatation.

**Results:**

Of a total of 169 patients with KD, 31 (18.3%) had maximal coronary Z-scores of ≥ + 2.5 during the acute KD phase, 16 (9.5%; male/female: 9/7) had coronary aneurysms at 1 month after KD onset, and 5 (3.0%) satisfied the definition of progressive coronary dilatation. Multivariate logistic regression analysis revealed that an initial maximal coronary Z-score of ≥ + 2.5 [odds ratio (OR): 5.24, 95% confidence interval (CI): 1.31–21.3, *P* = 0.020] and hypoalbuminemia (OR: 4.83, 95% CI: 1.11–20.9, *P* = 0.035) were independent risk factors for coronary aneurysms and were significantly associated with progressive coronary dilatation. However, the association between intravenous immunoglobulin unresponsiveness and the development of coronary aneurysms at 1 month after KD onset didn’t reach the level of significance (*P* = 0.058).

**Conclusions:**

In the present study, 3% (5/169) of patients with KD had progressive coronary dilatation, which was associated with persistent coronary aneurysms at 1 year after KD onset. Initial coronary dilatation and hypoalbuminemia were independently associated with the occurrence of progressive coronary dilatation. Therefore, such patients may require intensive cardiac monitoring and adjuvant therapies apart from immunoglobulin therapies.

## Background

Kawasaki disease (KD) is one of the most common forms of systemic vasculitis in children [1]. Even after intravenous immunoglobulin (IVIG) treatment, coronary arterial lesions (CALs) have been observed in 5%–20% of patients with KD during the acute stage [2–4]. In Taiwan and Japan, CALs have typically been classified into three subgroups (small [<4 mm], medium [4–8 mm], and giant [≥8 mm]) based on their diameters during the acute disease phase or at 1 month after disease onset [2, 4]. Coronary artery diameter-based severity is the most significant predictor of late coronary outcomes [5]. We observed that progressive coronary dilatation for at least 2 months was associated with worse late coronary outcomes in patients with KD having medium (4–8 mm) or giant (≥8 mm) aneurysms [6]. Several studies [7, 8] have evaluated KD-associated CALs by using body surface area-normalized coronary Z-scores and have demonstrated a significant reduction in coronary Z-scores from the initial values mostly in the first 2–3 months. McCrindle and his colleagues [8] reported some risk factors associated with a greater coronary Z-scores at any time, such as younger age and lower serum albumin levels [8]. However, whether the progressive increase of coronary Z-scores occurs in all KD patients, especially those without CAL or with small aneurysms at their acute phase, remains unclear.. Therefore, in the present study, we aimed to determine (1) the maximal coronary Z-score distributions in Taiwanese patients with KD at the acute, subacute, and convalescent phases; and (2) the risk factors for coronary aneurysms and progressive coronary dilatation in Taiwanese KD patients.

## Methods

This study was approved by the Institutional Review Board of National Taiwan University Hospital.

### Patients

In the present study, KD was diagnosed on the basis of the clinical criteria for KD [9]. Patients with KD who were admitted to our institution between January 2009 and December 2013 and were administered IVIG (2 g/kg × 1 day or 1 g/kg × 2 days) within 10 days after fever onset were enrolled in this study. However, patients with KD who had congenital heart disease were excluded from the study. The first day of illness was considered as the first day of fever. Patients with an axillary body temperature of <37.5 °C for >24 h were considered afebrile. The aspirin dosage was reduced to 5 mg/kg/day after defervescence. Medical records of all patients were reviewed, and the manifestations, symptoms, and laboratory data (including serum albumin levels and acute-phase reactants) were obtained as described in previous studies [5, 6]. Echocardiography was performed in all children during the febrile stage and the subacute phase (3–4 and 6–8 weeks) after fever onset, and the echocardiography frequency subsequently varied depending on CAL severity.

IVIG unresponsiveness was defined as the failure to respond to the initial IVIG dosage and the presence of persistent fever for >24 h or the development of KD-associated recrudescent fever after an afebrile period [1, 4, 5]. Patients unresponsive to IVIG were administered additional IVIG doses.

### Measurements

The coronary artery measurements were normalized to the body surface area using the established reference in Taiwanese children [10]. In the current study, we defined “coronary artery dilatation” as maximal Z-score > = +2.5 of any branch of coronary artery [1]. Only the coronary dilatation persisted for more than a month after disease onset were considered coronary aneurysms [5, 6]. The severity of coronary aneurysms was classified as small (+2.5 ≦ Z < +5.0), medium (+5.0 ≦ Z < 10) and giant (Z > = +10.0) [11]. CALs and the regression were diagnosed based on 2D echocardiography.

### Definition of progressive coronary dilatation

Progressive coronary dilatation was defined as the progressive dilatation of coronary arteries on three consecutive echocardiograms [6]. The coronary Z-score on the second echocardiogram had to be higher than that on the first echocardiogram, and the coronary Z-score on the third echocardiogram had to be 8% higher than that on the first echocardiogram. We defined progressive coronary dilatation based on the 8% increase criterion because a previous study [10] on Taiwanese coronary Z-scores showed interobserver differences of 7.1%, 5.8%, and 5.2% for the left main coronary artery, left anterior descending coronary artery, and right coronary artery, respectively. However, in the current study, the interobserver and intraobserver differences were 6.6% and 6.1%, respectively.

### Statistical analysis

Patient data are expressed as counts, percentages, medians with interquartile ranges (IQRs), and means (standard deviations). We used the independent Student *t* test and Fisher exact test for comparing continuous and categorical variables, respectively. Nonnormal variables were analyzed using the Mann–Whitney nonparametric test. A *P* value of <0.05 was considered statistically significant. The risk factors for coronary aneurysms derived from the univariate analysis were used in the subsequent logistic regression analysis. The logistic regression analysis was conducted to evaluate the potential risk factors for coronary aneurysms and progressive coronary dilatation. All analyses were performed using SPSS Statistics (Version 20.0. IBM Corp, Armonk, NY).

## Results

### Patient characteristics

Between 2009 and 2013, 175 patients with KD were admitted to our hospital. Of these 175 patients with KD, 6 (3.4%) were excluded because they received IVIG treatments beyond 10 days after fever onset. Finally, 169 patients with KD were enrolled in this study. The median age of these patients at the diagnosis of acute KD was 1.4 years (IQR: 8.1 months–2.5 years), and 99 (59%) were boys. The median duration of fever before the first course of IVIG treatment was 5 days (IQR: 4–6 days). Of the 169 KD patients, 138 (81.7%) were administered a single course of IVIG treatment (1 g/kg/day × 2 days or 2 g/kg/dose × 1 day), 20 (11.8%) received IVIG retreatment, and 11 (6.5%) did not receive IVIG treatment due to defervescence before IVIG administration. Moreover, the enrolled patients did not receive steroid therapies, and none of them died during the study period. Table 1 presents the characteristics of the patients with KD according to their maximal coronary Z-scores during the acute KD phase.

**Table 1:** Characteristics of patients with and without coronary dilatation during their acute KD phase

	All (169)	Normal (138)	Dilatation (31)	*P* value
Male gender	99 (59%)	81 (59%)	18 (58%)	0.949
Age (yr)	1.44 (0.67; 2.56)	1.46 (0.71; 2.74)	1.12 (0.36; 2.09)	0.063
IVIG unresponsive	20 (12%)	11 (8%)	9 (29%)	0.001
Days of fever before IVIG use	5 (5; 6)	5 (4.3; 6)	5 (5; 6.3)	0.518
Albumin (g/dL)	3.9 (3.5; 4.3)	4 (3.6; 4.3)	3.5 (3.2; 4.2)	0.03
AST (U/L)	38 (29; 66.5)	37 (29; 71.5)	39.5 (25.3; 56.3)	0.379
CRP (mg/dL)	7.23 (2.22; 14.15)	6.1 (3.26; 11.93)	14.05 (6.38; 18.29)	0.009
WBC (k/μL)	13.74 (10.32; 17.56)	13.61 (10.29; 16.94)	15.36 (10.74; 19.64)	0.418
Seg (%)	62.3 (51.3; 73.6)	62.9 (53.5; 73.9)	59 (49.7; 72)	0.391
Hb (g/dL)	11.1 (10.4; 12)	11.2 (10.5; 12)	10.9 (10.4; 11.8)	0.329
PLT (k/μL)	333 (260; 412)	328 (260; 405)	343 (253; 454)	0.285

Values are expressed as medians (IQRs) and percentages (%). *IVIG* intravenous immunoglobulin, *AST* Aspartate aminotransferase, *CRP* C-reactive protein, *WBC* White blood cell, *Seg* Neutro segment WBC, *Hb* hemoglobin, *PLT* platelet

### Echocardiography measurements

All enrolled patients underwent echocardiography before receiving IVIG treatment. The median maximal coronary Z-score of any coronary artery during the acute KD phase was 1.60 (IQR: +0.95 − +2.2). Table 1 shows the clinical and laboratory data of 31 and 138 patients with maximal coronary Z-scores of ≥ + 2.5 and <+2.5, respectively, during the acute KD phase. The coronary severity during the acute KD phase (before IVIG treatment) was associated with IVIG responsiveness, hypoalbuminemia, and C-reactive protein (CRP) levels. On the basis of the definition of coronary aneurysms (persistent coronary dilatation for more than 1 month after disease onset), 16 (9.5%) patients had coronary aneurysms (small, *n* = 14; medium, *n* = 2). At the end of follow-up (12 months after fever onset), four (25.0%) patients had small persistent coronary aneurysms. However, coronary aneurysms or progressive coronary dilatation was not observed in 11 patients who did not receive IVIG treatment due to defervescence within 10 days after KD onset.

### Risk factors associated with the coronary aneurysms

Univariate analysis revealed three potential risk factors associated with the coronary aneurysms, including initial maximal coronary Z-score of ≥ + 2.5, IVIG unresponsiveness, and serum albumin levels [Table 2].

**Table 2:** Univariate analysis of the risk factors for coronary aneurysms

	Coronary AN (*n* = 16)	Regression (*n* = 153)	*p*-value
Male gender	9 (56%)	90 (59%)	0.842
Age (yr)	0.99 (0.33; 1.86)	1.5 (0.69; 2.7)	0.059
Initial Z-score ≥ 2.5	10 (56%)	21 (14%)	<0.001
IVIG unresponsive	8 (50%)	12 (7.8%)	<0.001
Days of fever before IVIG use	5 (5; 7)	5 (4; 6)	0.346
Albumin (g/dL)	3.4 (2.85; 3.55)	4.0 (3.6; 4.3)	<0.001
AST (U/L)	45 (25; 87)	38 (29; 60.3)	0.885
CRP (mg/dL)	12.15 (6.35; 18.18)	6.68 (3,3; 13.37)	0.058
WBC (k/μL)	17.79 (9.52; 19.19)	13..65 (10.39; 16.94)	0.426
Seg (%)	63.1 (55.7; 77.5)	61.8 (51.1; 73.5)	0.721
Hb (g/dL)	10.8 (10.4; 11.5)	11.1 (10.45; 12)	0.307
PLT (k/μL)	399 (487; 886)	333 (262; 409)	0.965

Values are expressed as medians (IQRs) and percentages (%). *IVIG* Intravenous immunoglobulin, *AST* Aspartate aminotransferase, *CRP* C-reactive protein, *WBC* White blood cell, *Seg* Neutro segment WBC, *Hb* Hemoglobin, *PLT* Platelet

The median of serum albumin levels were significantly lower in the 16 KD patients with coronary aneurysms (3.4 g/dL, IQR: 2.85-3.55 g/dL) than in those without coronary aneurysms (4.0 g/dL, IQR: 3.6-4.3 g/dL; *P* < 0.001). When hypoalbuminemia was defined by serum albumin levels of <3.5 g/dL, it remained significantly associated with the development of coronary aneurysms (11/16 vs 25/153, *P* < 0.001) in this study.

Multivariate logistic regression analysis was conducted to evaluate the independent effects of an initial maximal coronary Z-score of ≥ + 2.5, hypoalbuminemia, and IVIG unresponsiveness on the development of coronary aneurysms in the 169 patients with KD. An initial maximal coronary Z-score of ≥ + 2.5 and hypoalbuminemia were independent risk factors for coronary aneurysms (odds ratio [OR] of initial maximal coronary Z-score of ≥ + 2.5: 5.24, 95% confidence interval (CI): 1.31–21.3, *P* = 0.020; OR of hypoalbuminemia: 4.83, 95% CI: 1.11–20.9, *P* = 0.035; OR of IVIG unresponsiveness: 4.63, 95% CI: 0.96–22.3, *P* = 0.058).

### Risk factors and implications of progressive coronary dilatation

**Fig. 1:**
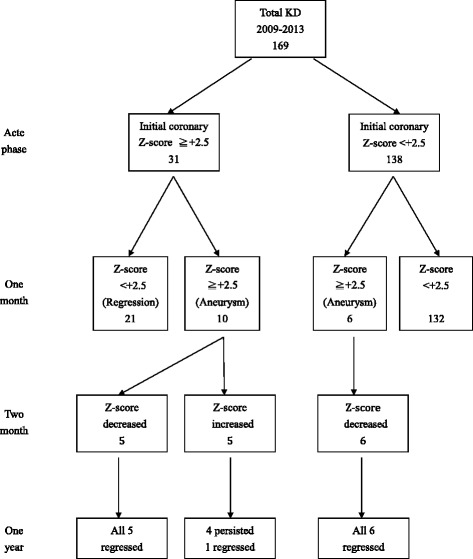
Flowchart of coronary follow-up of 169 patients with KD

The coronary Z-scores of six patients increased from <+2.5 initially to ≥ + 2.5 at 1 month after KD onset; however, none of these patients exhibited any further increase in their coronary Z-scores on subsequent echocardiographic examinations (Fig. 1). Of the 10 patients with persistent coronary aneurysms at 1 month after KD onset, 5 showed more increased coronary Z-scores at 2 months after KD onset (Fig. 1), thus satisfying the criteria of progressive coronary dilatation on three consecutive echocardiograms. Of the five patients with progressive coronary dilatation, four had persistent coronary aneurysms even at 1 year after KD onset. Compared with the remaining 11 patients without progressive coronary dilatation, 4 patients with progressive coronary dilatation had a higher probability of persistent coronary aneurysms at 1 year after KD onset (0/11 vs 4/5, *P* = 0.003).

Of the 31 patients with KD initial coronary Z-scores of ≥ + 2.5, 5 (16.1%) had progressive coronary dilatation on three consecutive echocardiograms. However, none of the remaining 138 patients with initial coronary Z-scores of <+2.5 showed such progression (5/31 vs 0/138; *P* = 2 × 10^−4^). Furthermore, of the 36 KD patients with hypoalbuminemia, 5 developed progressive coronary dilatation. Moreover, the patients with normal serum albumin levels did not develop progressive coronary dilatation (*P* = 3 × 10^−4^). The incidence of progressive coronary dilatation did not differ significantly between the IVIG-responsive and IVIG-unresponsive patients with KD (3/138 vs 2/20, *P* = 0.11). To avoid the interaction of risk factors, multivariate logistic regression analysis was conducted to determine the risk factors for progressive coronary dilatation. The results confirmed that an initial maximal coronary Z-score of ≥ + 2.5 (OR: 10.94, 95% CI: 1.14–104.91, *P* = 0.038) and hypoalbuminemia (OR: 9.25, 95% CI: 1.001–88.93, *P* = 0.049) were independent risk factors for progressive coronary dilatation in the patients with KD. However, IVIG unresponsiveness was not significantly associated with progressive coronary dilatation (OR: 1.85, 95% CI: 0.26–13.14, *P* = 0.54)

## Discussion

In this study, based on serial echocardiographic measurements, we recognized two independent clinical characteristics (initial maximal coronary Z-score of ≥ + 2.5 and hypoalbuminemia during the acute KD phase) that were significantly associated with coronary aneurysms at 1 month after KD onset as well as progressive coronary dilatation. Progressive coronary dilatation has been insufficiently explored before. McCrindle et al. [8] examined coronary artery involvement in children with KD and observed that if the maximal coronary Z-score is <+2.5 on the initial echocardiogram, it might increase above +2.5 on subsequent echocardiograms in 6% of patients, which is consistent with our study findings (6/138, 4.3%). Moreover, our recent study reported progressive coronary dilatation for at least 2 months in KD patients with medium (25.5%) or giant (48.1%) aneurysms [6]. Furthermore, the current study confirmed that progressive coronary dilatation can occur in KD patients with small aneurysms (3/14, 21.4%), though with a less probability. In 5 (31.3%) of the 16 patients with coronary aneurysms in the current study, the aneurysms enlarged on three consecutive echocardiographic examinations. Moreover, these five patients were more likely to have persistent coronary aneurysms for more than 1 year compared with those without progressive coronary dilatation (4/5 vs 0/11, *P* = 0.003). These findings support our previous observation that progressive coronary dilatation is associated with worse late coronary outcomes [6].

A recent study reported that 81% of patients with KD who eventually developed coronary aneurysms showed coronary abnormalities on their initial echocardiograms [12]. Studies have proposed the use of adjuvant therapies with agents such as atorvastatin [13], steroids [14, 15], and dalteparin [16] to ameliorate the CALs of patients with KD during the acute KD phase. Friedman et al. [17] demonstrated that the rate of coronary aneurysm regression was significantly higher in patients with KD receiving IVIG and adjunctive medications than in those not receiving such medications (91% vs 68%, *P* = 0.02). Our present study showed that patients with KD having initial maximal coronary Z-scores of ≥ + 2.5, particularly those with hypoalbuminemia, are susceptible to progressive coronary dilatation. However, additional studies are warranted to elucidate whether IVIG and adjuvant therapies can promote the regression of coronary aneurysms and prevent their progressive dilatation in patients with KD.

Crystal et al. showed that greater coronary Z-scores over the complete study period were significantly associated with greater initial coronary Z-scores [18], which supports the findings of our studies. Previous studies have identified a few risk factors for coronary dilatation or aneurysmal formation [19–21], including late IVIG treatment, IVIG unresponsiveness, and several clinical biomarkers, such as serum albumin levels and CRP levels. In the current study, we further demonstrated hypoalbuminemia during the acute KD phase was also significantly associated with progressive coronary dilatation. In addition, of the 16 patients with coronary aneurysms at 1 month after KD onset (Fig. 1), 5 showed more increased coronary Z-scores at 2 months after KD onset. Furthermore, All of the five patients with progressive coronary dilatation had hypoalbuminemia (<3.5 g/dL, 100%), indicating that the incidence of hypoalbuminemia was higher in the aforementioned patients than in the remaining 11 patients with coronary aneurysms (*P* = 0.012). These findings may indicate that patients with KD who develop hypoalbuminemia during the acute KD phase, particularly those with coronary Z-scores of ≥ + 2.5, are susceptible to progressive coronary dilatation and may require closer cardiac monitoring and more aggressive treatments using agents such as statins [13] and steroids [14, 15].

However, the reason for the association of serum albumin levels with coronary aneurysms and progressive coronary dilatation remains unclear. Terai et al. [22] reported that IVIG-unresponsive patients with KD had higher vascular endothelial growth factor levels, which might lead to vascular leakage, decreased serum albumin levels, and pericardial effusion. Therefore, hypoalbuminemia is most likely caused by vascular inflammation and thus is associated with coronary aneurysms and progressive dilatation in patients with KD.

Previous studies have reported CRP as one of the risk factors for IVIG unresponsiveness [21] and an independent risk factor for initial coronary dilatation [23] and giant aneurysms [24] in patients with KD. However, in the current study, the CRP levels were not significantly associated with coronary aneurysms (*P* = 0.058) or progressive coronary dilatation (*P* = 0.54). Our earlier study revealed that low-grade inflammation was associated with persistent CALs in patients with KD [25]. Therefore, we investigated the association between progressive coronary dilatation and the changes in inflammatory biomarkers (CRP levels, white cell count, and neutrophil percentages) during the acute febrile and subacute phases. However, none of the changes in the three inflammatory biomarkers were associated with progressive coronary dilatation. Therefore, additional studies are warranted to elucidate the effects of CRP and other KD-associated inflammatory biomarkers, such as interleukin-4 [26] and interleukin-6 [27], on early and late CALs in patients with KD.

### Limitations

Our study has several limitations. First, this study was conducted in a single tertiary medical center in Taiwan, which may have resulted in selection bias. Second, this study had a retrospective design, and a limited number of patients were enrolled. Third, information bias may have existed, because ultrasound technicians were not blinded to tentative diagnoses. Finally, we did not analyze the socioeconomic factors, febrile days on initial IVIG, and unmeasured laboratory data, such as alanine aminotransferase and bilirubin levels, which were potential confounders in the current study. Large, prospective cohort studies are necessary to reduce the influence of potential confounders.

## Conclusions

Coronary artery dilatation with an initial maximal coronary Z-score of ≥ + 2.5 and hypoalbuminemia during the acute KD phase are independent risk factors for coronary artery aneurysms and progressive coronary dilatation in the subacute KD phase. These simple indicators may help clinicians in identifying high-risk KD children who may have coronary aneurysms and progressive coronary dilatation and require intensive monitoring and additional therapies.

## Abbreviations

CAL: Coronary arterial lesions
CI: Confidence interval
CRP: C-reactive protein
IQR: Interquartile range
IVIG: Intravenous immunoglobulin
KD: Kawasaki disease
OR: Odds ratio
